# Hepatic Localization of Macrophage Phenotypes during Fibrogenesis and Resolution of Fibrosis in Mice and Humans

**DOI:** 10.3389/fimmu.2014.00430

**Published:** 2014-09-08

**Authors:** Leonie Beljaars, Marlies Schippers, Catharina Reker-Smit, Fernando O. Martinez, Laura Helming, Klaas Poelstra, Barbro N. Melgert

**Affiliations:** ^1^Department of Pharmacokinetics, Toxicology and Targeting, University of Groningen, Groningen, Netherlands; ^2^Botnar Research Centre, Nuffield Department of Orthopaedics, Rheumatology and Musculoskeletal Sciences, University of Oxford, Oxford, UK; ^3^Institute for Medical Microbiology, Immunology and Hygiene, Technische Universität München, Munich, Germany

**Keywords:** cirrhosis, fibrosis, resolution, M1, M2, IRF-5, IL-12, TGM-2

## Abstract

Macrophages have been found to both promote liver fibrosis and contribute to its resolution by acquiring different phenotypes based on signals from the micro-environment. The best-characterized phenotypes in the macrophage spectrum are labeled M1 (classically activated) and M2 (alternatively activated). Until now the *in situ* localization of these phenotypes in diseased livers is poorly described. In this study, we therefore aimed to localize and quantify M1- and M2-dominant macrophages in diseased mouse and human livers. The scarred collagen-rich areas in cirrhotic human livers and in CCl4-damaged mouse livers contained many macrophages. Though total numbers of macrophages were higher in fibrotic livers, the number of *parenchymal* CD68-positive macrophages was significantly lower as compared to normal. Scar-associated macrophages were further characterized as either M1-dominant (IRF-5 and interleukin-12) or M2-dominant (CD206, transglutaminase-2, and YM-1) and significantly higher numbers of both of these were detected in diseased livers as compared to healthy human and mouse livers. Interestingly, in mouse, livers undergoing resolution of fibrosis, the total number of CD68^+^ macrophages was significantly lower compared to their fibrotic counterparts. M2-dominant (YM-1) macrophages were almost completely gone in livers undergoing resolution, while numbers of M1-dominant (IRF-5) macrophages were almost unchanged and the proteolytic activity (MMP9) increased. In conclusion, this study shows the distribution of macrophage subsets in livers of both human and murine origin. The presence of M1- and M2-dominant macrophages side by side in fibrotic lesions suggests that both are involved in fibrotic responses, while the persistence of M1-dominant macrophages during resolution may indicate their importance in regression of fibrosis. This study emphasizes that immunohistochemical detection of M1/M2-dominant macrophages provides valuable information in addition to widely used flow cytometry and gene analysis.

## Introduction

Chronic injury of the liver leads to induction of fibrogenic processes that ultimately can progress to cirrhosis, a state in which excessive extracellular matrix deposition hampers normal liver functions. Hepatic stellate cells (HSC) are regarded as the principal cells that are involved in scar tissue deposition (1, 2). Recent studies indicate that the role of Kupffer cells has been underestimated in fibrogenesis and consequently hepatic macrophages have gained more attention recently (3–7). Kupffer cells are well-known producers of reactive oxygen species, cytokines and chemokines, that perpetuate hepatic inflammatory responses, and of matrix-degrading enzymes. In addition, these macrophages can phagocytose micro-organisms, apoptotic cells, and cellular debris generated during tissue injury and remodeling. Duffield et al. (8) clearly showed that Kupffer cells exert different, even opposing roles during various stages of liver fibrosis. They showed that macrophage activities during the injury phase were predominantly associated with promotion of matrix deposition and HSC activities, while during recovery macrophages were associated with enhanced resolution and higher production of matrix metalloproteinases (MMPs) (8, 9). These diverse roles may indicate that activated macrophages differentiate into diverse phenotypes during various stages of liver disease.

Activated macrophages are described to polarize into different phenotypes depending on signals they receive from their environment. Many types can be distinguished, and the most-used, but rather simplified, classification system discerns classically activated macrophages (also called M1) and alternatively activated macrophages (also called M2) (10–12). In fact, these phenotypes represent their dominant appearance in a wide spectrum of overlapping activation types. Other M2-like transitional phenotypes have been described as well, but to date these have been difficult to distinguish from M2 macrophages *in situ* in tissues due to lack of phenotype-specific markers (6, 13–15). In general, M1-dominant macrophages have enhanced microbicidal and tumoricidal capacity and secrete high levels of pro-inflammatory cytokines like interleukin-12 (IL-12). M1-dominant macrophages can also inhibit fibrotic activities of fibroblasts by releasing antifibrogenic or fibrolytic factors such as MMPs (16, 17). M2-dominant macrophages, activated by interleukin-4 and interleukin-13, are associated with increased fibrogenesis, tissue remodeling, and angiogenesis (17–19). *In vitro*, Song et al. (17) showed that the M2-dominant macrophages produce profibrogenic factors like platelet-derived growth factor-BB (PDGFBB) and transforming growth factor-β (TGFbeta) and that these M2-dominant cells increase collagen production and proliferation of fibroblasts. Although M2 macrophages are predominantly considered to be pro-fibrotic, they are also associated with anti-fibrotic properties, which may be explained by the different and overlapping M2 phenotypes that exist (5, 11). For instance, M2 macrophages can also aid resolution of fibrosis by phagocytizing apoptotic cells and matrix components via mannose and scavenger receptors (20–22). In addition, Pesce et al. (23) showed that arginase-1 expressing M2 cells were related to suppression rather than induction of fibrosis.

Thus far, most of the knowledge generated about the different macrophage subsets is derived from *in vitro* studies, from flow cytometry analyses of isolated liver macrophages (6), and from gene analysis of liver homogenates (24). Although these techniques generate useful quantitative information, histological detection of macrophages gives unique and additional information with regard to their tissue localization without selection due to isolation limitations or with minor risk of missing changes because other cells express the same markers, such as observed in tissue homogenates (25).

How the different phenotypes are distributed in diseased liver tissue is still largely unexplored. Therefore, we aimed to illustrate, using immunohistochemical techniques, how different macrophage phenotypes are distributed *in situ* during fibrogenic responses and resolution of fibrosis using the general M1 and M2 classification as a starting point. Of the markers commonly used, we chose IL-12 and IRF-5 as markers for the M1-dominant subtype (26). Inducible nitric oxide synthase (iNOS), another commonly used M1 marker, was not chosen because its dominant expression in hepatocytes would make distinguishing neighboring iNOS expressing macrophages difficult (27, 28). To detect M2 polarization, we used upregulation of the mannose receptor (MRC1; also known as CD206), transglutaminase-2 (TGM-2), and chitinase-like secretory protein YM-1 (mouse only) (29–32). TGM-2 was recently identified as a new human and murine M2 marker (33). The commonly used M2 marker arginase could not be used for reasons similar to iNOS (27).

## Materials and Methods

### Animals

Male mice (BALB/c, ±25 g) were obtained from Harlan (Zeist, The Netherlands) and housed in a temperature-controlled room with 12 h light/dark regimen. The animal experiments were approved by the Institutional Animal Care and Use Committee (IACUC) of the University of Groningen (The Netherlands) and were performed according to strict governmental and international guidelines on animal experimentation.

### Animal models

#### Chronic liver injury (fibrosis) model

Mice received twice-weekly intraperitoneal injections of CCl4 for 4 or 8 weeks. The dose of CCl4 was gradually increased (diluted in olive oil; week 1: 0.5 ml/kg, week 2: 0.8 ml/kg, week 3–8: 1 ml/kg). Mice were sacrificed after 4 or 8 weeks reflecting early and advanced fibrosis, respectively.

#### Resolution model

Mice received CCl4 for 4 weeks (with increasing CCl4 doses as described in the previous section). After 4 weeks, CCl4 administration was stopped and the mice were allowed to recover for a week after which they were sacrificed (*n* = 6 per group).

### Human livers

Residual human liver tissue samples were obtained from the Department of Hepato-Pancreato-Biliary Surgery and Liver Transplantation [University Medical Center Groningen (UMCG), the Netherlands]. At the UMCG, all patients eligible for organ transplantation are asked to sign a general consent form for the use of left-over body material (after diagnostic procedures) for research purposes. The experimental protocols were approved by the Medical Ethical Committee of the UMCG (Groningen) and the anonymized tissue samples were used according to Dutch guidelines. Normal human liver tissue (*n* = 7) was obtained from residual liver tissue from donor livers discarded for transplantation because of technical reasons. Cirrhotic human liver tissue (*n* = 6) was obtained from patients undergoing liver transplantation. Indications for transplantation were a.o. primary sclerosing cholangitis (PSC), primary biliary cirrhosis (PBC), congenital cirrhosis, and Wilson’s cirrhosis. Although all human liver material is anonymized, some available patient characteristics are listed below (see Table 1).

**Table 1:** **Available patient characteristics of the used human livers**.

Patient characteristics	Normal livers	Cirrhotic livers
	*N* = 7	*N* = 6
Age (years)	41 (10–57)	49 (35–66)
Gender	*N* = 4: F	*N* = 3: F
	*N* = 2: M	*N* = 1: M
	*N* = 1: not known	*N* = 2: not known

### Tissue processing

Tissue specimens from at least three different mouse liver lobes were snap frozen in isopentane (−80°C) for immunohistochemical analysis, or in liquid nitrogen for western blot analysis.

A wedge (10–60 g) of freshly obtained human liver was cut, perfused with cold University of Wisconsin organ storage solution (DuPont Critical Care, Waukegan, IL, USA) immediately after resection, and pieces were snap frozen in isopentane (−80°C).

### Immunohistochemical analysis

Acetone-fixed cryostat sections (4 μm) were stained according to standard immunohistochemical procedures with 3-amino-9-ethyl-carbazole to detect expression of relevant markers (32). Sections were incubated with the primary antibody for 1 h. Primary antibodies to detect fibrotic extracellular matrix (polyclonal goat anti-collagen type I from Southern Biotech), macrophages [mouse anti-human CD68 (DAKO), monoclonal rat anti-mouse CD68 (AbD Serotec, Düsseldorf, Germany), and polyclonal rabbit anti-human CD68 (Santa Cruz Biotechnology)], M1 macrophages [polyclonal rabbit anti-human and mouse IRF-5 (Protein Tech, Manchester, UK), goat polyclonal anti-human IL-12 p40 antibody (ThermoScientific), and goat polyclonal anti-human and mouse MMP9 (Santa Cruz)], and M2 macrophages [polyclonal goat anti-mouse chitinase 3-like/ECF-L (YM-1; R&D), rabbit anti-human TGM-2 (AbD Serotec) and CD206 (rat anti-mouse CD206 and mouse anti-human CD206) both from BioLegends (ITK Diagnostics, Uithoorn, The Netherlands)] were used. Staining of CD68 was quantified by image analysis with Cell^∧^D analysis program (Olympus, Zoetermeer, The Netherlands).

To detect co-localization, we used double-staining techniques with peroxidase and AEC (red) and alkaline phosphatase and Naphtol AS-MX phosphate/Fast Blue BB (blue) (34). Double stainings for IRF-5, IL-12, and CD206 were visualized with NovaRed (red) and BCIP/NBT (blue) from Vector Laboratories.

### Western blot analysis

Tissues samples were homogenized on ice in cold RIPA buffer [50 mM Tris-HCl, 150 mM NaCl, 0.1% SDS, 0.1% Igepal in 0.5% sodium deoxycholate with one tablet of protease inhibitor cocktail and one tablet of phosphatase inhibitor (Roche Diagnostics, Mannheim, Germany)] and lysates were centrifuged for 1 h (13,000 rpm, 4°C). The supernatants were stored at −80°C. Total protein (100 μg) from each sample was applied on SDS-PAGE (10%), transferred to polyvinylidene difluoride membranes, and incubated overnight at 4°C with the indicated primary antibodies. After washing and incubation with secondary horseradish peroxidase-coupled antibodies, the protein bands were visualized with ECL (Perkin-Elmer, Groningen, The Netherlands) and quantified by G-Box (Syngene, Cambridge, UK).

In order to quantify the marker of interest, we corrected the expression with the expression of the housekeeping protein GADPH (for human samples) or β-actin (for mouse samples).

### Statistical analysis

Results are expressed as means ± SEM. All data were analyzed with the Mann–Whitney *U* test (Graph Pad software). Differences were considered significant at *p* < 0.05.

## Results

### Localization of CD68-positive macrophages in human and mouse fibrotic livers

**Figure 1:**
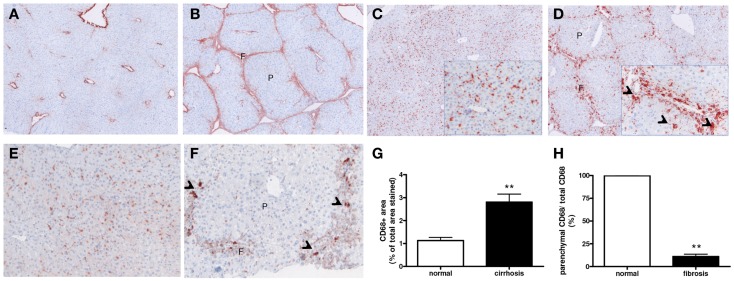
**Expression and localization of macrophages in normal and fibrotic mouse livers (8 weeks CCL4)**. Immunohistochemical analysis shows increased extracellular matrix deposition [collagen type I **(A,B)**] and presence of macrophages [CD68 **(C–F)**] in fibrotic **(B,D,F)** as compared to healthy livers **(A,C,E)**. Note the increased size of certain CD68-positive macrophages in the fibrotic areas **(F)** in the CCL4 livers [arrow heads in insert **(C,F)**]. **(G,H)** Image analysis of CD68 staining. While the total area of CD68+ cells was increased in fibrotic livers, a significantly lower CD68-stained area was found in the parenchyma *(p)* of fibrotic livers as compared to normal. Magnifications: 40× **(A–D)**, 100× **(E,F)**, and 200× (*inserts*). f, Fibrotic matrix; *p*, liver parenchyma. *N* = 6/group. **p < 0.01.

After chronic CCl4 damage (8 weeks CCL4), pericentral necrosis led to wound-healing responses with influx of myofibroblasts and increased collagen deposition (Figures 1A,B) in mouse livers. As compared to normal livers, a higher number of CD68-positive cells was found in fibrotic livers, and these macrophages predominantly concentrated in scars during advanced fibrosis (Figures 1C,D,G). Furthermore, several scar-associated macrophages differed in appearance from macrophages in normal livers, even some resembled giant cells (Figures 1D,F). Remarkably, significantly less staining for CD68^+^ cells was found in the parenchymal areas of fibrotic livers as compared to normal (Figures 1E–H).

**Figure 2:**
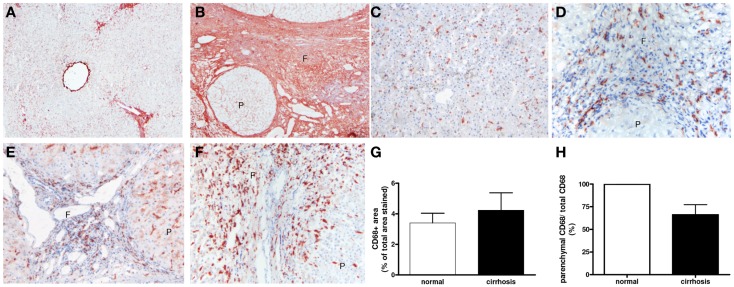
**Expression and localization of macrophages in normal and cirrhotic human livers**. Immunohistochemical analysis shows enhanced deposition of the extracellular matrix protein collagen type I **(A,B)** and presence of macrophages [CD68 **(C–F)**] in normal **(C)** cirrhotic livers of various origins [**(D)** PBC **(E)** PSC and **(F)** congenital cirrhosis]. Note the abundant presence of macrophages in the collagenous fibrotic bands **(F)**. **(G,H)** Image analysis of CD68 staining in human livers. Reduced CD68 staining was found in the parenchymal area (*p*) of human cirrhotic livers as compared to normal. Magnifications: 40× **(A,B)** and 100× **(C–F)**. f, fibrotic matrix; *p*, liver parenchyma. *N* = 5 cirrhotic livers, *N* = 6 normal livers.

Collagen deposition was also greatly increased in end-stage human cirrhotic livers (Figures 2A,B), and a similar hepatic distribution of macrophages as in mice was found (Figure 2). Macrophages (CD68^+^) were prominently present in cirrhotic scars irrespective of the origin of cirrhosis (Figures 2D–F). The total number of CD68^+^ cells was somewhat, though not significantly, higher in cirrhotic than normal livers (Figure 2G). Again, a trend toward less staining for CD68 was found in the parenchymal areas of cirrhotic livers as compared to healthy livers (Figure 2H).

### M1-dominant macrophages in mouse and human livers

**Figure 3:**
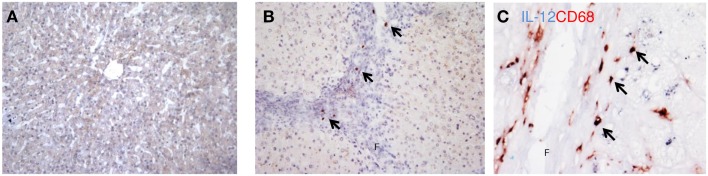
**Localization of IL-12 (M1) in cirrhotic human livers**. Immunohistochemical staining for IL-12p40 in cirrhotic human livers **(B)**, while no staining was observed in normal livers **(A)**. **(C)** Co-localization of IL-12 and CD68. Arrows indicate co-localization, f, fibrotic matrix. Magnifications: 100× **(A,B)** and 400× **(C)**. *N* = 5 cirrhotic livers, *N* = 6 normal livers.

**Figure 4:**
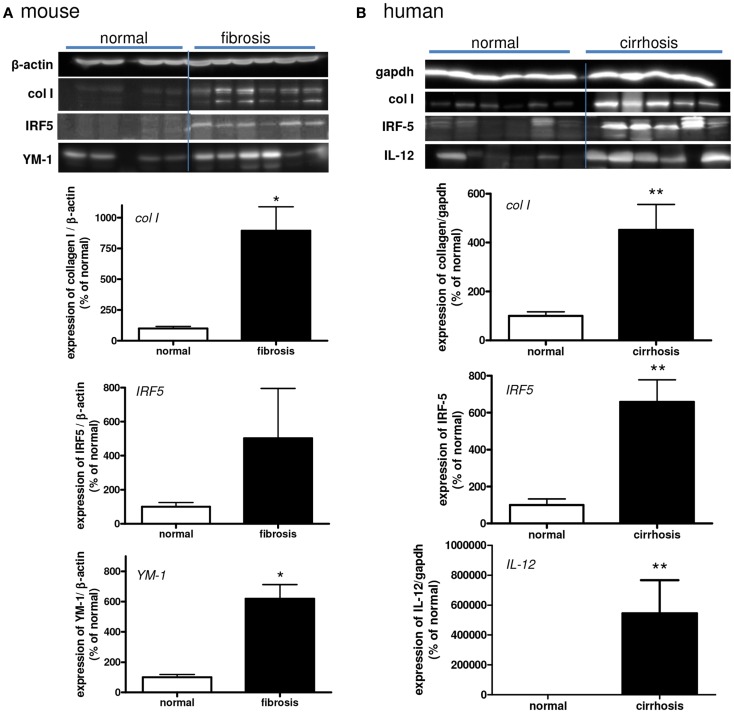
**Western blot analysis of the expressions of collagen type I, IRF-5 (M1), IL-12 (M1), and YM-1 (M2) in normal and fibrotic mouse (A) and human (B) livers**. *p < 0.05. *N* = 5 cirrhotic human livers, *N* = 6 normal human livers, *N* = 4 normal mouse livers, *N* = 6 fibrotic mouse livers.

Interleukin-12, a major cytokine produced by classically activated macrophages, was used as an immunohistochemical marker to detect the M1-dominant subset in the liver (Figure 3). In human livers, higher numbers of IL-12-positive cells were detected in cirrhotic human livers (Figure 3B) as opposed to barely detectable IL-12 staining in healthy livers (Figure 3A). This increased expression was confirmed by western blot analysis (Figure 4B). IL-12 positive cells were found solely in the cirrhotic collagen bands. The staining for IL-12 co-localized completely with CD68, but only a minor fraction of the CD68 population was positive for IL-12 (Figure 3C).

**Figure 5:**
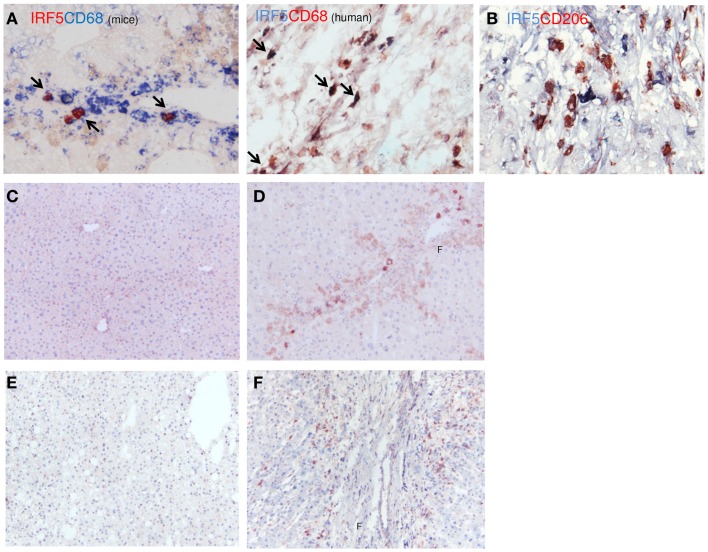
**Localization of IRF-5 (M1) in mouse and human livers**. **(A)** Co-localization of IRF-5 and CD68 in mouse and human livers. **(B)** Double-staining for IRF-5 (blue staining) and CD206 (red staining) showed no co-localization. **(C,D)** Immunohistochemical staining of IRF-5 in normal mice livers **(C)** and in livers after chronic CCl4 damage **(D)**. **(E,F)** IRF-5 staining of normal **(E)** and cirrhotic **(F)** human livers. f, fibrotic matrix. Magnifications: 100× **(C–F)** and 400× **(A,B)**. *N* = 5 cirrhotic human livers, *N* = 6 normal human livers, *N* = 4 normal mouse livers, *N* = 6 fibrotic mouse livers.

IRF-5 was also used to identify M1 macrophages in human and mouse livers (Figure 5). IRF-5 staining co-localized completely with CD68 in livers of both species (Figure 5A). Similar to IL-12, only a subset of the total number of macrophages expressed IRF-5. To prove the phenotype-specificity of this M1 marker, we performed double-immunostainings of IRF-5 and the M2 marker CD206 in human livers and found little to no co-localization (Figure 5B). Microscopic analysis showed that IRF-5 staining was almost absent in normal mouse and human livers (Figures 5C,E). In advanced fibrotic mouse livers, the staining was present in cells residing in the scarred areas (Figure 5D). Similarly, in human livers IRF-5 staining was also predominantly found in cells of the septa (Figure 5F). Western blot analysis of liver homogenates revealed a significantly higher expression of IRF-5 in diseased mouse and human livers as compared to healthy livers (Figures 4A,B).

### M2-dominant macrophages in mouse and human livers

**Figure 6:**
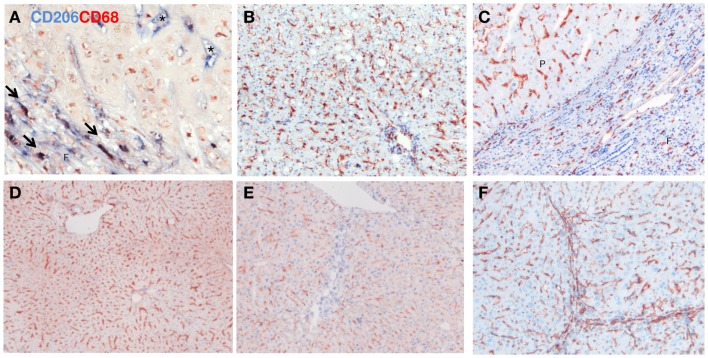
**Immunohistochemical staining for CD206 (MCR-1; mannose receptor) in human (A–C) and mouse (D,E) normal (B,D) and cirrhotic (C,E) livers**. **(A)** Co-localization of CD206 (blue staining) and CD68 (red staining). Arrows indicate co-localization, asterisks indicate endothelial staining of CD206. **(F)** Immunohistochemical staining for CD31 in fibrotic mouse livers illustrating staining of sinusoidal endothelial cells. Magnifications: 100× **(B–F)** and 400× **(A)**. *N* = 5 cirrhotic human livers, *N* = 6 normal human livers, *N* = 4 normal mouse livers, *N* = 6 fibrotic mouse livers.

**Figure 7:**
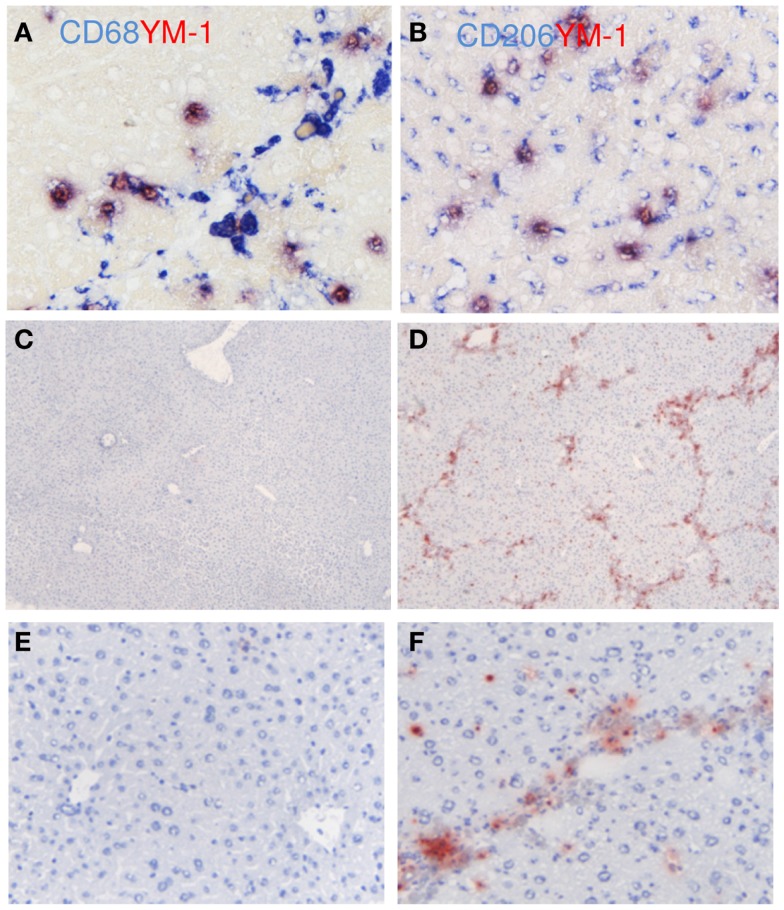
**Localization of YM-1 (M2) in mouse livers**. **(A)** Co-localization of YM-1 (red staining) and CD68 (blue staining). **(B)** Co-localization of YM-1 (red staining) and CD206 (blue staining). **(C–F)** Immunohistochemical localization of YM-1 in livers of normal mice **(C,E)** and in advanced fibrosis **(D,F)**. Magnifications: 40× **(C,D)**, 200× **(E,F)**, and 400× **(A,B)**. *N* = 4 normal mouse livers, *N* = 6 fibrotic mouse livers.

**Figure 8:**
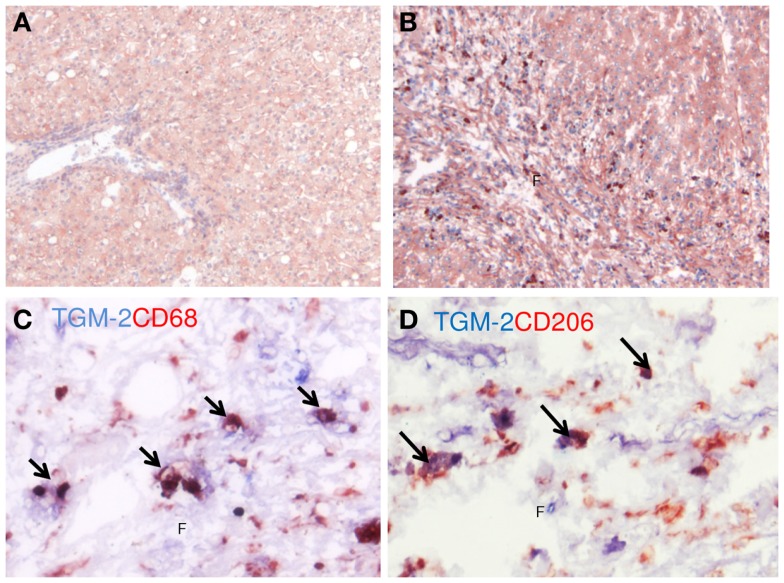
**Localization TGM-2 (M2) in human livers**. **(A,B)** Immunohistochemical localization of TGM-2 in normal **(A)** and cirrhotic **(B)** human livers. Note the presence of the strongly stained cells in the fibrotic matrix **(F)**. **(C)** Co-localization of TGM-2 (blue staining) and CD68 (red staining). **(D)** Co-localization of TGM-2 (blue staining) and CD206 (red staining). Arrows indicate co-localization. Magnifications: 100× **(A,B)** and 400× **(C,D)**. *N* = 5 cirrhotic human livers, *N* = 6 normal human livers.

Subsequently, we studied the hepatic distribution of alternatively activated macrophages (Figures 6–8). CD206 is a well-known marker for both mouse and human M2-dominant macrophages. CD206/CD68 double-positive cells were present in fibrotic livers and were predominantly found in scars (Figure 6A). In addition to this, CD206 staining was present in liver parenchyma and this staining most likely reflected expression of CD206 on sinusoidal endothelial cells [identified with CD31 (Figure 6F)]. The pronounced endothelial staining of CD206 complicates interpretation of analyzes of whole tissue homogenates (like western blot and mRNA expression analyses) used in the macrophage field. Microscopic evaluation of sections stained for both CD206 and CD68 indicated that double-positive cells were more frequent in fibrotic liver than in normal livers, whereas western blot analysis revealed reduced expression in both human and mouse fibrotic livers (data not shown).

YM-1 was used as another M2 marker for mouse livers (Figure 7). Expression of YM-1 is restricted to mice and can therefore not be used for human liver tissue (29). YM-1 co-localized with CD68 and with CD206 (Figures 7A,B). All cells that expressed YM-1 were positive for CD68 and CD206, but not all CD68-positive cells stained positive for YM-1. The hepatic expression of YM-1 was clearly higher after chronic CCl4 damage as demonstrated by immunohistochemical staining (Figures 7C–F) and western blot analysis (Figure 4A). YM-1 was present in the fibrotic collagenous bands of the CCL4-damaged livers.

The recently described M2 marker TGM-2 (33) was also used to identify M2-dominant macrophages in human livers (Figure 8). Immunohistochemical staining for TGM-2 resulted in staining of the parenchymal area of normal livers, mostly staining hepatocytes, but in cirrhotic livers additional strong positive cells were found in septa (Figures 8A,B). TGM-2 staining present in scars co-localized with CD68 (Figure 8C) and with CD206 (Figure 8D) confirming presence of TGM-2 in hepatic M2-dominant macrophages that accumulate in these areas. As with iNOS, arginase-1, and CD206, quantitative evaluation of TGM-2 was confounded by its high expression in hepatocytes. We could not detect differences between normal and cirrhotic livers (data not shown).

### M1- and M2-dominant macrophages in a mouse model of resolution

**Figure 9:**
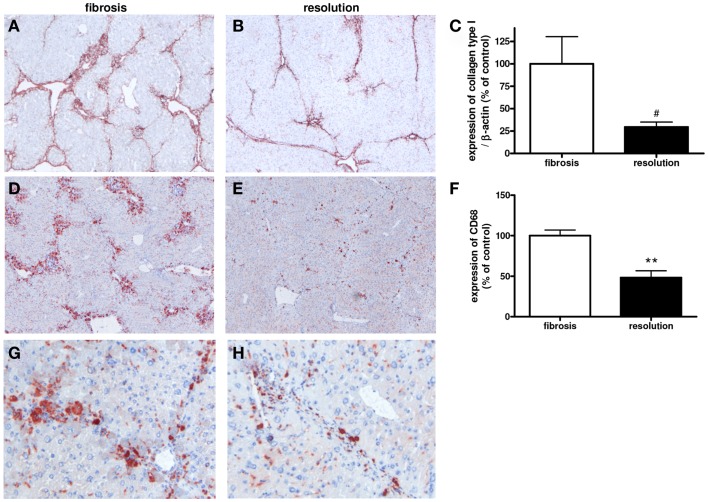
**Immunohistochemical and western blot analysis of the hepatic expressions of collagen type I (A–C) and macrophages [CD68 (D–H)] in fibrosis [4 weeks of CCl4 in mice (A,D,G)] and in livers undergoing resolution [after cessation of CCl4 administration, resolution (B,E,H)]**. **p < 0.01, ^#^*p* < 0.1. Magnifications: 40× **(A–E)** and 200× **(G,H)**. *N* = 6/group.

Cessation of fibrosis-inducing agents induces reversal of the fibrotic process (35). This is also apparent in our mouse model with lower hepatic collagen type I in livers of mice in which CCl4 administration was stopped versus their fibrotic equivalents (Figures 9A–C). Since macrophages are important during resolution (8, 9), we studied the localization and numbers of macrophage phenotypes in these two groups of mice. Expression of CD68 was significantly lower in livers undergoing resolution as compared to their fibrotic counterparts (Figures 9D–H).

**Figure 10:**
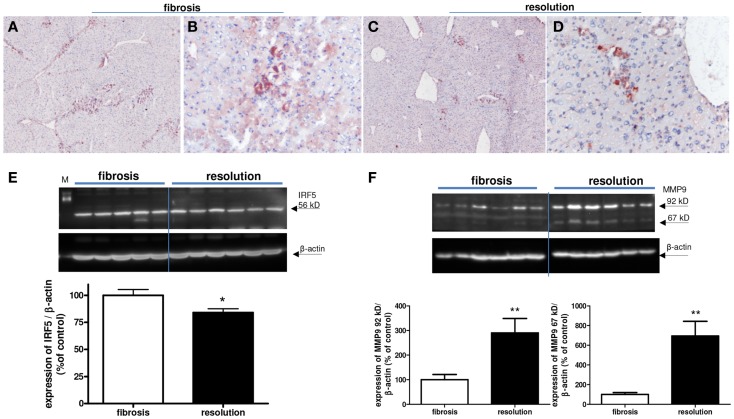
**Expressions of IRF-5 and MMP9 (M1) in fibrotic mouse livers [4 weeks CCl4 (A,B)] and in fibrotic livers undergoing resolution [after cessation of 4 weeks of CCl4 administration (C,D)]**. Immunohistochemical pictures demonstrate an overview [**(A,C)** magnification 40×] and close up [**(B,D)** magnification 200×]. **(E)** Western blot quantification of hepatic IRF-5 expression in fibrosis versus resolution group, and **(F)** western blot quantification of MMP9 expression in fibrosis versus resolution group. A 92 kDa pro-form and a 67 kDa processed form of MMP9 is significantly increasingly expressed in livers undergoing resolution. *p < 0.05, **p < 0.01. *N* = 6/group.

**Figure 11:**
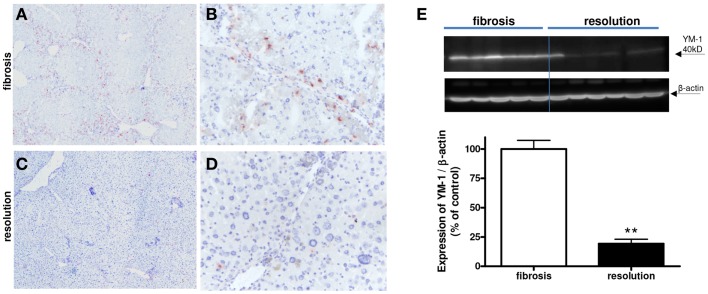
**Expressions of YM-1 (M2) in fibrotic mouse livers [4 weeks CCl4 (A,B)] and in fibrotic livers undergoing resolution [after cessation of CCl4 administration (C,D)]**. Immunohistochemical pictures demonstrate an overview [**(A,C)** magnification 40×] and close up [**(B,D)** magnification 200×]. **(E)** Western blot quantification of YM-1 expression in fibrosis versus resolution. **p < 0.01. *N* = 6/group.

We detected a slightly reduced expression of IRF-5 with both immunohistochemical and western blot analysis (Figure 10). However, a clear difference in the number of M2-dominant macrophages was found (Figure 11). YM-1 staining was abundantly present in fibrotic livers, but in livers undergoing resolution this M2 marker was almost completely gone. Western blot analysis revealed a reduction of 81 ± 8% in YM-1 expression during resolution (Figure 11E).

Since the number of IRF-5^+^ (M1-dominant) macrophages was almost unchanged in fibrotic livers compared to livers undergoing resolution, we measured MMP expression as a functional read out of the presence of these macrophages. M1-dominant cells are known to express MMP9 (17, 21, 36, 37) and western blot analysis of these livers revealed significantly higher expression of MM9 92 kDa, which is known as pro-MMP9, and its processed form (67 kDa MMP9) in the livers undergoing resolution (Figure 10F).

## Discussion

Recently, activation of macrophages into different phenotypes has been subject of study in various diseases including in liver diseases. Almost all knowledge obtained thus far is derived from *in vitro* studies or from FACS or PCR analyses of tissues. These *in vitro* studies have been essential to discover markers to distinguish the various macrophages phenotypes and to identify the specific activities of these subsets. How these *in vitro*-generated phenotypes relate to macrophages *in situ* is largely unexplored. In this study, results were obtained from the CCL4 mouse model at several time points in disease progression (reflecting early and advanced fibrosis) and resolution. Although we are aware that more time points in this mouse model can support broader conclusions, our outcomes with regard to the presence and localizations of the various macrophage phenotypes are first steps toward understanding the dynamics of macrophage phenotypes in relation to localization. A major advantage of our studies is the verification of mouse data in samples of human liver disease. The fact that we find similar distributions of macrophage phenotypes in end-stage disease of a number of different etiologies may point at converging disease mechanisms irrespective of cause.

We used many commonly used markers M1- and M2-dominant phenotypes and found that not all of them can be used reliably for liver tissue. With the ones that can be used, we demonstrated that M1- and M2-dominant subsets are localized side by side in scars of human and mouse cirrhotic livers. Although M1 and M2 markers can be expected to be present on the same cell, based on the theory of overlapping spectra of macrophage subsets (13–15), with our markers (IRF-5 and CD206), we found little to no co-localization. We showed that IL-12 and IRF-5 are useful immunohistochemical markers for M1-dominant macrophages in liver tissue (both mouse and human) and YM-1 for M2-dominant macrophages in murine liver tissue. CD206 and TGM-2 can be useful for immunohistochemistry of M2-dominant macrophages in human liver tissue, but are much less specific and therefore are hard to quantify. Furthermore, in fibrotic livers undergoing resolution we found that M2-dominant macrophages (YM-1 positive cells) disappeared, while M1-dominant macrophages (IRF-5 positive cells) persisted in the scarred areas producing MMPs.

Interleukin-12 and IRF-5 were used to identify the classically activated macrophages in fibrotic livers. Krausgruber et al. (26) showed high expression of IRF-5 in human M1 macrophages in culture, while M2 and non-activated macrophages did not express IRF-5. We now show that IRF-5 can be used to identify a subset of macrophages *in vivo* in human and mouse livers. Our study clearly demonstrates that M1-dominant macrophages (CD68/IRF-5^+^ cells) are significantly increased in diseased livers as compared to normal. IRF-5^+^ cells are located in fibrous septa in advanced fibrosis. These localizations may correspond with reported *in vitro* M1 activities such as production of pro-inflammatory cytokines and chemokines (5, 11, 17). The observation that M1-dominant macrophages are still present in livers undergoing resolution might be related to their ability to produce MMPs (12, 16, 17, 37). Classical activation of macrophages *in vitro* resulted in higher expression of MMP7 and MMP9 and both may be necessary in the collagenous scars for removal of collagen fibers. Indeed, in our studies we detected higher MMP9 expression in fibrotic livers undergoing resolution. In addition, Fallowfield et al. (9) demonstrated higher hepatic MMP13 expression by scar-associated macrophages in CCL4-damaged livers and it was found that resolution of CCl4-induced fibrosis was retarded in MMP13-deficient mice. However, macrophage phenotypes in these scars were not further characterized. We now show with our localization studies that during fibrogenesis scar-associated macrophages are both of M1- and M2-dominant phenotype, while during resolution the scar-associated macrophages are predominantly M1 cells. It therefore appears that M1 macrophages may also be responsible for the MMP13 production that is necessary for resolution. Co-localization studies with IRF-5 and MMP13 may provide additional insights.

To identify M2-dominant macrophages, we started with the well-known marker CD206 (mannose receptor, MCR-1) (11). While in many organs M2-dominant macrophages specifically express CD206, in livers CD206 expression is found in macrophages as well as in sinusoidal endothelial cells, making quantitative interpretations difficult. In addition, we therefore used the well-known M2-selective marker YM-1, which does not have this disadvantage. However, this marker is only present in rodents (29) and cannot be used for human tissues. TGM-2 is a novel marker for M2 macrophages recently described by us in lungs (33). The advantage of TGM-2 is that this marker is conserved in mice and humans. TGM-2 is a multifunctional enzyme involved in transamidation and cross-linking of proteins. It is also linked to apoptosis, cellular differentiation, and matrix stabilization (38–40). In liver, Popov et al. (41) showed that TGM-2 is enhanced in mice with CCl4-induced fibrosis, but they found no relationship between TGM-2 and stabilization of fibrotic matrix. However, TGM-2 expression was not related to macrophage activities. Although the hepatic expression is not limited to macrophages, as can be seen in Figure 8, TGM-2 staining in the scar-associated macrophages in cirrhotic livers is much stronger than in other hepatic cells. Therefore, this marker can be used for immunohistochemical stainings but quantification using western blot or PCR will not yield useful results. To summarize, using a combination of the markers CD206, YM-1, and TGM-2, we are able to show that M2-dominant macrophages are present in scar tissue during hepatic fibrogenesis. We now show that TGM-2 is co-expressed in CD68^+^ and in CD206^+^ cells in fibrotic septa in human and mouse livers, confirming its presence in M2-dominant hepatic macrophages.

This study clearly shows the presence of M1- and M2-dominant macrophages side by side in fibrotic lesions in human and mouse livers, indicating that apparently both are necessary in fibrotic responses. At least two questions remain: (1) where do these macrophages come from, meaning are they derived from incoming monocytes and are thus bone marrow-derived or do they develop from tissue-resident Kupffer cells that are embryonic in origin (42). Unfortunately, our study cannot answer this question, as there are no markers discovered yet that can reliably distinguish bone marrow-derived from embryonic macrophages. Previous studies showed that monocytes do infiltrate the liver during fibrogenesis and resolution and also that Kupffer cells do proliferate during injury (43, 44). Understanding the dynamics of all these different macrophages during fibrogenesis/resolution and their interactions is a subject of intense research interest. (2) How these macrophage phenotypes interact with each other and with other resident cells to enhance or dissolve fibrosis. Song et al. (17) showed that M2-dominant macrophages increased the proliferation index and collagen synthesis of co-cultivated WI-38 fibroblasts, while M1-dominant macrophages markedly reduced collagen production by these cells. Most *in vitro* studies suggest that M2 activation results in enhanced fibrogenesis, while M1 activation inhibits fibrogenesis through antifibrogenic or fibrolytic factors. Just recently, Lopez-Navarrette (18) showed the importance of M2-dominant macrophages in promoting fibrogenesis in a CCl4-induced model of liver fibrosis in which Kupffer cells were stimulated to polarize to an M2-dominant phenotype after hepatic inoculation of *Taenia crassiceps* larvae. Our results also suggest a more pro-fibrotic character of M2-dominant macrophages, because M2 markers were present in fibrotic lesions in human and mouse livers, but were nearly absent in the livers during resolution of fibrosis.

Recently, Ramachandran et al. (6) suggested a restorative role for macrophages during resolution of fibrosis after cessation of CCL4 intoxications using flow cytometry. The persistence of M1-dominant macrophages during resolution in our studies indicates that this restorative phenotype may have a more M1 bias. M1-dominant macrophages have been reported to be major producers of various MMPs and MMP-producing macrophages were previously reported to be present during liver regeneration in mice (3, 9, 16). However, M2-dominant macrophages can also express MMPs [most notably MMP12 (45)] and were found to be important cells for efferocytosis and phagocytosis of matrix debris (16, 21, 46–48). These characteristics of M2-dominant cells may also be necessary during the resolution phase. The reason we do not see M2-dominant macrophages anymore in our resolution model may be caused by the fact that the resolution is ongoing (based on reduced hepatic collagen deposition) and these functions of M2-dominant macrophages may have less important. Studying macrophage phenotype localizations at more time points during resolution may shed more light on the specific dynamics of the macrophage phenotypes during resolution.

In conclusion, using a set of established as well as recently identified markers we now clearly show local accumulation of both M1- and M2-dominant macrophages in fibrotic septa of mouse and human end-stage cirrhotic livers. This provides a basis for further exploring the different activities of these various macrophage phenotypes during liver fibrosis and resolution of fibrosis. The observation that during liver remodeling M1-dominant macrophages may persist and M2-dominant macrophages may disappear indicates that different combinations of M1 versus M2-dominant macrophages may play a key role in fibrogenesis and resolution. Manipulation of their balance may therefore be of therapeutic value.

## Conflict of Interest Statement

The authors declare that the research was conducted in the absence of any commercial or financial relationships that could be construed as a potential conflict of interest.

## References

[1] Pinzani M Rombouts K Colagrande S Fibrosis in chronic liver diseases: diagnosis and management. J Hepatol (2005) 42(Suppl 1):S22–36 10.1016/j.jhep.2004.12.008 15777570

[2] Friedman SL Evolving challenges in hepatic fibrosis. Nat Rev Gastroenterol Hepatol (2010) 7(8):425–36 10.1038/nrgastro.2010.97 20585339

[3] Ramachandran P Iredale JP Macrophages: central regulators of hepatic fibrogenesis and fibrosis resolution. J Hepatol (2012) 56(6):1417–9 10.1016/j.jhep.2011.10.026 22314426

[4] Heymann F Trautwein C Tacke F Monocytes and macrophages as cellular targets in liver fibrosis. Inflamm Allergy Drug Targets (2009) 8(4):307–18 10.2174/187152809789352230 19534673

[5] Wynn TA Barron L Macrophages: master regulators of inflammation and fibrosis. Semin Liver Dis (2010) 30(3):245–57 10.1055/s-0030-1255354 20665377 PMC2924662

[6] Ramachandran P Pellicoro A Vernon MA Boulter L Aucott RL Ali A Differential Ly-6C expression identifies the recruited macrophage phenotype, which orchestrates the regression of murine liver fibrosis. Proc Natl Acad Sci U S A (2012) 109(46):E3186–95 10.1073/pnas.1119964109 23100531 PMC3503234

[7] Sica A Invernizzi P Mantovani A Macrophage plasticity and polarization in liver homeostasis and pathology. Hepatology (2014) 59(5):2034–42 10.1002/hep.26754 24115204

[8] Duffield JS Forbes SJ Constandinou CM Clay S Partolina M Vuthoori S Selective depletion of macrophages reveals distinct, opposing roles during liver injury and repair. J Clin Invest (2005) 115(1):56–65 10.1172/JCI22675 15630444 PMC539199

[9] Fallowfield JA Mizuno M Kendall TJ Constandinou CM Benyon RC Duffield JS Scar-associated macrophages are a major source of hepatic matrix metalloproteinase-13 and facilitate the resolution of murine hepatic fibrosis. J Immunol (2007) 178(8):5288–95 10.4049/jimmunol.178.8.5288 17404313

[10] Gordon S Martinez FO Alternative activation of macrophages: mechanism and functions. Immunity (2010) 32(5):593–604 10.1016/j.immuni.2010.05.007 20510870

[11] Mantovani A Sica A Sozzani S Allavena P Vecchi A Locati M The chemokine system in diverse forms of macrophage activation and polarization. Trends Immunol (2004) 25(12):677–86 10.1016/j.it.2004.09.015 15530839

[12] Martinez FO Sica A Mantovani A Locati M Macrophage activation and polarization. Front Biosci (2008) 13:453–61 10.2741/2692 17981560

[13] Mosser DM Edwards JP Exploring the full spectrum of macrophage activation. Nat Rev Immunol (2008) 8(12):958–69 10.1038/nri2448 19029990 PMC2724991

[14] Mantovani A Biswas SK Galdiero MR Sica A Locati M Macrophage plasticity and polarization in tissue repair and remodelling. J Pathol (2013) 229(2):176–85 10.1002/path.4133 23096265

[15] Mills CD Ley K M1 and M2 macrophages: the chicken and the egg of immunity. J Innate Immun (2014). 10.1159/000364945 25138714 PMC4429858

[16] Huang WC Sala-Newby GB Susana A Johnson JL Newby AC Classical macrophage activation up-regulates several matrix metalloproteinases through mitogen activated protein kinases and nuclear factor-kappaB. PLoS One (2012) 7(8):e42507 10.1371/journal.pone.0042507 22880008 PMC3411745

[17] Song E Ouyang N Horbelt M Antus B Wang M Exton MS Influence of alternatively and classically activated macrophages on fibrogenic activities of human fibroblasts. Cell Immunol (2000) 204(1):19–28 10.1006/cimm.2000.1687 11006014

[18] Lopez-Navarrete G Ramos-Martinez E Suarez-Alvarez K Aguirre-Garcia J Ledezma-Soto Y Leon-Cabrera S Th2-associated alternative Kupffer cell activation promotes liver fibrosis without inducing local inflammation. Int J Biol Sci (2011) 7(9):1273–86 10.7150/ijbs.7.1273 22110380 PMC3221364

[19] Gibbons M MacKinnon A Ramachandran P Dhaliwal K Duffin R Phythian-Adams A Ly6Chi monocytes direct alternatively activated profibrotic macrophage regulation of lung fibrosis. Am J Respir Crit Care Med (2011) 184(5):569–81 10.1164/rccm.201010-1719OC 21680953

[20] Wynn TA Ramalingam TR Mechanisms of fibrosis: therapeutic translation for fibrotic disease. Nat Med (2012) 18(7):1028–40 10.1038/nm.2807 22772564 PMC3405917

[21] Boorsma CE Draijer C Melgert BN Macrophage heterogeneity in respiratory diseases. Mediators Inflamm (2013) 769214:19 10.1155/2013/769214 PMC3600198 23533311

[22] Lopez-Guisa JM Cai X Collins SJ Yamaguchi I Okamura DM Bugge TH Mannose receptor 2 attenuates renal fibrosis. J Am Soc Nephrol (2012) 23(2):236–51 10.1681/ASN.2011030310 22095946 PMC3269177

[23] Pesce JT Ramalingam TR Mentink-Kane MM Wilson MS El Kasmi KC Smith AM Arginase-1-expressing macrophages suppress Th2 cytokine-driven inflammation and fibrosis. PLoS Pathog (2009) 5(4):e1000371 10.1371/journal.ppat.1000371 19360123 PMC2660425

[24] Louvet A Teixeira-Clerc F Chobert MN Deveaux V Pavoine C Zimmer A Cannabinoid CB2 receptors protect against alcoholic liver disease by regulating Kupffer cell polarization in mice. Hepatology (2011) 54(4):1217–26 10.1002/hep.24524 21735467

[25] Randolph GJ Proliferating macrophages prevail in atherosclerosis. Nat Med (2013) 19(9):1094–5 10.1038/nm.3316 24013746

[26] Krausgruber T Blazek K Smallie T Alzabin S Lockstone H Sahgal N IRF5 promotes inflammatory macrophage polarization and TH1-TH17 responses. Nat Immunol (2011) 12(3):231–8 10.1038/ni.1990 21240265

[27] Uhlen M Oksvold P Fagerberg L Lundberg E Jonasson K Forsberg M Towards a knowledge-based human protein atlas. Nat Biotechnol (2010) 28(12):1248–50 10.1038/nbt1210-1248 21139605

[28] Vos TA Van Goor H Tuyt L De Jager-Krikken A Leuvenink R Kuipers F Expression of inducible nitric oxide synthase in endotoxemic rat hepatocytes is dependent on the cellular glutathione status. Hepatology (1999) 29(2):421–6 10.1002/hep.510290231 9918918

[29] Raes G Van den Bergh R De Baetselier P Ghassabeh GH Scotton C Locati M Arginase-1 and Ym1 are markers for murine, but not human, alternatively activated myeloid cells. J Immunol (2005) 174(11):6561 10.4049/jimmunol.174.11.6561 15905489

[30] Raes G De Baetselier P Noel W Beschin A Brombacher F Hassanzadeh Gh G Differential expression of FIZZ1 and Ym1 in alternatively versus classically activated macrophages. J Leukoc Biol (2002) 71(4):597–602 11927645

[31] Van Gorp H Delputte PL Nauwynck HJ Scavenger receptor CD163, a Jack-of-all-trades and potential target for cell-directed therapy. Mol Immunol (2010) 47(7–8):1650–60 10.1016/j.molimm.2010.02.008 20299103

[32] Melgert BN ten Hacken NH Rutgers B Timens W Postma DS Hylkema MN More alternative activation of macrophages in lungs of asthmatic patients. J Allergy Clin Immunol (2011) 127(3):831–3 10.1016/j.jaci.2010.10.045 21167569

[33] Martinez FO Helming L Milde R Varin A Melgert BN Draijer C Genetic programs expressed in resting and IL-4 alternatively activated mouse and human macrophages: similarities and differences. Blood (2013) 121(9):e57–69 10.1182/blood-2012-06-436212 23293084

[34] Beljaars L Molema G Weert B Bonnema H Olinga P Groothuis GM Albumin modified with mannose 6-phosphate: a potential carrier for selective delivery of antifibrotic drugs to rat and human hepatic stellate cells. Hepatology (1999) 29(5):1486–93 10.1002/hep.510290526 10216133

[35] Iredale JP Models of liver fibrosis: exploring the dynamic nature of inflammation and repair in a solid organ. J Clin Invest (2007) 117(3):539–48 10.1172/JCI30542 17332881 PMC1804370

[36] Murray PJ Wynn TA Protective and pathogenic functions of macrophage subsets. Nat Rev Immunol (2011) 11(11):723–37 10.1038/nri3073 21997792 PMC3422549

[37] Hanania R Sun HS Xu K Pustylnik S Jeganathan S Harrison RE Classically activated macrophages use stable microtubules for matrix metalloproteinase-9 (MMP-9) secretion. J Biol Chem (2012) 287(11):8468–83 10.1074/jbc.M111.290676 22270361 PMC3318683

[38] Belkin AM Extracellular TG2: emerging functions and regulation. FEBS J (2011) 278(24):4704–16 10.1111/j.1742-4658.2011.08346.x 21902810 PMC3228878

[39] Fesus L Piacentini M Transglutaminase 2: an enigmatic enzyme with diverse functions. Trends Biochem Sci (2002) 27(10):534–9 10.1016/S0968-0004(02)02182-5 12368090

[40] Fesus L Szondy Z Transglutaminase 2 in the balance of cell death and survival. FEBS Lett (2005) 579(15):3297–302 10.1016/j.febslet.2005.03.063 15943974

[41] Popov Y Sverdlov DY Sharma AK Bhaskar KR Li S Freitag TL Tissue transglutaminase does not affect fibrotic matrix stability or regression of liver fibrosis in mice. Gastroenterology (2011) 140(5):1642–52 10.1053/j.gastro.2011.01.040 21277850 PMC3374132

[42] Guilliams M Ginhoux F Jakubzick C Naik SH Onai N Schraml BU Dendritic cells, monocytes and macrophages: a unified nomenclature based on ontogeny. Nat Rev Immunol (2014) 14(8):571–8 10.1038/nri3712 25033907 PMC4638219

[43] Liaskou E Zimmermann HW Li KK Oo YH Suresh S Stamataki Z Monocyte subsets in human liver disease show distinct phenotypic and functional characteristics. Hepatology (2013) 57(1):385–98 10.1002/hep.26016 22911542 PMC4194426

[44] Tacke F Zimmermann HW Macrophage heterogeneity in liver injury and fibrosis. J Hepatol (2014) 60(5):1090–6 10.1016/j.jhep.2013.12.025 24412603

[45] Nelson MP Christmann BS Dunaway CW Morris A Steele C Experimental pneumocystis lung infection promotes M2a alveolar macrophage-derived MMP12 production. Am J Physiol Lung Cell Mol Physiol (2012) 303(5):L469–75 10.1152/ajplung.00158.2012 22773692 PMC3468423

[46] Madala SK Pesce JT Ramalingam TR Wilson MS Minnicozzi S Cheever AW Matrix metalloproteinase 12-deficiency augments extracellular matrix degrading metalloproteinases and attenuates IL-13-dependent fibrosis. J Immunol (2010) 184(7):3955–63 10.4049/jimmunol.0903008 20181883 PMC3175622

[47] Dalli J Serhan CN Specific lipid mediator signatures of human phagocytes: microparticles stimulate macrophage efferocytosis and pro-resolving mediators. Blood (2012) 120(15):e60–72 10.1182/blood-2012-04-423525 22904297 PMC3471524

[48] Morimoto K Janssen WJ Terada M Defective efferocytosis by alveolar macrophages in IPF patients. Respir Med (2012) 106(12):1800–3 10.1016/j.rmed.2012.08.020 22999220 PMC4030720

